# Cluster analysis for symptomatic management of *Neisseria gonorrhoea* and *Chlamydia trachomatis* in sexually transmitted infections related clinics in China

**DOI:** 10.3389/fpubh.2022.1005481

**Published:** 2022-11-17

**Authors:** Ning Ning, Rongxing Weng, Chunlai Zhang, Lizhang Wen, Honglin Wang, Jianbin Ye, Jing Li, Xiangsheng Chen, Yumao Cai

**Affiliations:** ^1^Shenzhen Center for Chronic Disease Control, Shenzhen, China; ^2^Shantou University Medical College, Shantou University, Shantou, China; ^3^Chinese Academy of Medical Sciences, Institute of Dermatology, Peking Union Medical College, Nanjing, China; ^4^National Center for STD Control, China Center for Disease Control and Prevention, Nanjing, China

**Keywords:** sexually transmitted infections, genital chlamydia trachomatis infection, *Neisseria gonorrhoeae*, symptomatic management, associated factors

## Abstract

**Objective:**

This study aimed to perform a cluster analysis of symptoms linked with *Neisseria gonorrhoeae* (NG) and *Chlamydia trachomatis* (CT) and to identify which cluster of symptoms was associated with a higher risk of NG and CT.

**Study design:**

From 15 April to 16 May 2018, a cross-sectional study was conducted, and patients attending sexually transmitted infections (STI) related clinics were recruited from 22 medical institutions in six districts of Shenzhen city.

**Methods:**

A structured questionnaire was used to collect social-demographic information as well as STI symptoms, and urine samples were collected for nucleic acid detection. Cluster analysis and logistic regression were applied.

**Results:**

Among 8,207 participants, the prevalence of CT and NG infection was 9.04% (742/8,207) and 2.36% (194/8,207), respectively. Among male outpatients, four clusters with distinct symptomatic patterns were identified. Unmarried, having casual sexual partners in the past 6 months, cluster 2 (OR = 6.70, 95% CI = 3.36–13.35) and cluster 4 (OR = 24.53, 95% CI = 12.96–46.44) were risk factors associated with NG infection. Unmarried, cluster 2 (OR = 2.54, 95% CI = 1.83–3.53) and cluster 4 (OR = 3.31, 95% CI = 2.37–4.61) were risk factors associated with CT infection. Among female outpatients, five clusters with distinct symptomatic patterns were identified. Aged 24 years or below and cluster 3 (OR = 3.68, 95% CI = 1.61–8.39) were risk factors associated with NG infection. Aged 24 years or below, unmarried, having a high school/secondary technical school education, and having junior high school or below education were risk factors associated with CT infection.

**Conclusion:**

The cluster of symptoms integrated into risk assessment for CT and NG infections suggests a new strategy of symptomatic management. Healthcare providers in STI clinics and resource-limited places may use this strategy to identify more potential patients and deliver adequate, acceptable, and equitable STI care for outpatients with a high risk of STI.

## Introduction

*Chlamydia trachomatis* (CT) and *Neisseria gonorrhoeae* (NG) are the most common sexually transmitted infections (STIs) worldwide. In 2016, the WHO estimated that there were 376.4 million new global cases of four common STIs: chlamydia (127.2 million cases), gonorrhoea (86.9 million cases), trichomoniasis (156 million cases), and syphilis (6.3 million cases) (1). Untreated NG and CT could lead to serious complications in women, including pelvic inflammatory disease, tubal infertility, and obstetric complications, and in men, including epididymitis and reactive arthritis (2). With this great burden of NG and CT, efforts are needed to find and promote appropriate strategies.

Symptomatic management of STI has been widely used and is a useful strategy to assess the risk of STI, with three main processes including identifying consistent groups of symptoms and signs, using the available flowcharts to provide treatment, and taking care of the most serious organisms responsible for producing the syndrome (3). As symptomatic management is a simple and practical strategy, it is still the standard of treatment when laboratory diagnosis is unavailable or when it takes a few days to obtain results in most resource-limited settings (4). Previous studies showed that symptomatic management can provide better treatment for patients with STIs at a significantly lower cost and significantly reduce the prevalence of syphilis, chancroid, gonorrhoeae, and bacterial vaginosis infection (5, 6). Symptomatic management for urethral discharge and genital ulcer disease in men had high sensitivity and accuracy, while in women, symptomatic management for abnormal vaginal discharge, genital ulcers, and genital warts was controversial (4, 7, 8). It may also lead to overtreatment or drug resistance in some subjects. In many diseases, syndrome cluster analysis was used to combine symptoms and identify those clusters associated with a higher risk of infection (9, 10).

Although the prevalence of CT infection in China was high (11, 12), CT infection is currently one of the STIs being monitored in China and is not included in notifiable communicable diseases. Aside from a pilot screening program in Shenzhen (13), the rate of routine screening is low because of the presence of asymptomatic patients, laboratory capability (e.g., primary care medical institutions with limited resources), and cost constraints, especially in rural areas with poor conditions. In China, 36.11% of people live in rural areas (14), and medical resources are limited in most rural areas (15). Therefore, symptomatic management could be a good strategy to find more infected people and provide timely treatment. Recently, an updated version of guidelines for the symptomatic management of symptomatic sexually transmitted infections was published by WHO (16), and there is a need to explore the use of symptomatic management from guidelines for the management of symptomatic sexually transmitted infections in the Chinese context. To better identify patients, a cluster analysis of symptoms linked with NG and CT was used in this study to identify which clusters of symptoms were associated with a higher risk of NG and CT and provide new strategies for symptomatic management in China.

## Materials and methods

### Sampling methods and recruitment

The multi-stage stratified sampling method was used to select study units. Six administrative districts in the city were randomly selected. In each district, the top four hospitals with the most reported cases of STI infection in 2017 were selected, except for one district with only two hospitals. A total of 22 medical institutions from Nanshan, Luohu, Bao'an, Longgang, Yantian, and Longhua districts were selected. From 15 April to 16 May 2018, the first 15 eligible attendees were recruited daily in the departments of dermatology, urology, and other STI-related departments of each medical institution (such as MSM counseling clinics). This study's inclusion criteria were as follows: (1) aged between 18 and 49 years; (2) having engaged in sexual behavior; and (3) not using antibiotics in the previous 2 weeks. All eligible patients were only recruited. All participants provided informed consent before the study. This study has been approved by the Ethical Review Committee of the Shenzhen Center for Chronic Disease Control (Approval No. 20180206).

### Patients recruitment and management

Patients were investigated by medical staff who had received professional training. The questionnaire included age, marital status, education, history of STI testing, casual sex partners in the last 3 months, and NG/CT-related symptoms. After laboratory testing, patients who were positive for CT and/or NG infections were informed of the testing results to ensure timely treatment. Positive cases were suggested to be retested for the infection 3 months after treatment, and partner notification and following treatment of their positive sexual partners were also recommended.

### Specimen collection and laboratory testing

A labeled Roche Cobas ^®^ urine collection tube was used to collect 15–30 ml of urine, which was transferred to each hospital's laboratory for preservation. At the central laboratory, DNA was extracted and purified from the urine specimens by the automated magnetism nucleic acid isolation method using the MagNA Pure 96 System (Roche, Switzerland). The extracted DNA was further evaluated for CT and NG based on polymerase chain reaction (PCR) of the Cobas 48001 System (Roche, Switzerland) using the Cobas1 4800 CT/NG Amplification/Detection Kit. Laboratory performance was run according to standard operating procedures (SOPs). A CT or NG infection was defined as having a positive PCR for CT or NG accordingly.

### Statistical analyses

Data were double-blind entered, and Epidata 3.0 was used to create the database. The investigator deleted duplicate data by assigning unique study numbers to each participant. First, cluster analysis was conducted on 10 symptoms of men and women, respectively. Second, we adopted a multivariate logistic regression model with forward selection method, defining NG or CT infection as the dependent variable and age, marital status, education, history of STI testing, casual sexual partners in the last 3 months, and NG/CT-related symptom clusters as independent variables. All data analysis was performed on SAS 8.0. All tests were two-tailed, and *P* < 0.05 was considered statistically significant.

## Result

A total of 8,207 participants completed the questionnaire and provided a urine sample, with 2,564 (31.01%) men and 5,703 (68.99%) women. Of all participants, 85.70% were aged 24 years or below, 65.50% were married, 31.80% were in junior high school or below, 29.09% were in high school/secondary technical school, 35.91% had casual sexual partners in the last 3 months, 89.50% had no history of STI testing, and 40.68% was asymptomatic. Totally, 2.36% (194/8,207) and 9.04% (742/8,207) of the participants were diagnosed with gonorrhea and chlamydia infections, respectively.

### Men

#### Cluster analysis in men

Among male attendees, four clusters with distinct symptomatic patterns and one group of asymptomatic patients were identified. The frequency of gonorrhea and chlamydia in each cluster is shown in Table 1. In this group, 5.88% (149/2,536) and 10.69% (271/2,536) of the participants were diagnosed with gonorrhea and chlamydia, respectively. The asymptomatic group was the largest, accounting for 50.12% (1271/2,536) of the total cohort. Cluster 1 consisted of 3.35% (85/2,536) of the entire cohort, including (1) scrotum swelling or pain and (2) epididymis pain/swelling/tenderness, and 2.35 and 8.24% of the participants were diagnosed with gonorrhea and chlamydia. Cluster 2 consisted of 23.54% (597/2,536) of the entire cohort, including (1) frequent urination/urgency/urodynia/itching, (2) balanitis, and (3) inguinal lymph node enlargement, and 5.70 and 14.41% of the participants were diagnosed with gonorrhea and chlamydia, respectively. Cluster 3 consisted of 3.55% (90/2,536) of the entire cohort, including (1) genital/perianal watery blisters or pustules and (2) genital/perianal ulcers, and 3.33 and 7.78% of the participants were diagnosed with gonorrhea and chlamydia, respectively. Cluster 4 consisted of 19.44% (493/2,536) of the entire cohort, including (1) urethral serous discharge, (2) urethral purulent discharge, and (3) genital/perianal warts, and 19.88 and 18.86% of the participants were diagnosed with gonorrhea and chlamydia, respectively. Besides, the symptom clusters of the MSM were also obtained, which showed a similar pattern.

**Table 1:** Symptom clusters of the respondents in the men cohort (*N* = 2,536).

**Symptoms**	**Asymptomatic** ***n =* 1,271**	**Cluster 1** ***n =* 85**	**Cluster 2** ***n =* 597**	**Cluster 3** ***n =* 90**	**Cluster 4** ***n =* 493**
Urethral serous discharge					192
Urethral purulent discharge					164
Frequent urination/urgency/ urodynia/itching			535		
Scrotum swelling or pain		58			
Epididymis pain, swelling and/or tenderness		32			
Genital/Perianal watery blisters or pustules				57	
Genital/Perianal ulcers				49	
Genital/Perianal warts					166
Balanitis			65		
Inguinal lymph node enlargement			2		
NG positive	12	2	34	3	98
CT positive	78	7	86	7	93

#### Factors associated with NG/CT infection in men

In univariate analyses, six variables were associated with NG/CT infection in men at *P* < 0.05 (shown in Tables 2, 3). Results from the multivariate logistic regression model suggested that the following factors were significantly associated with NG infection: unmarried (AOR = 1.89, 95% CI = 1.32–2.70), having casual sexual partners in the last 3 months (AOR = 2.03, 95% CI = 1.37–2.99), cluster 2 (AOR = 6.70, 95% CI = 3.36–13.35), and cluster 4 (AOR = 24.53, 95% CI = 12.96–46.44). Using cluster 2 and cluster 4 as an indicator of NG can find 88.59% (132/149) NG positive cases, but the number of tests accounted for only 42.98% (1,090/2,536) of total male participants. Results from the multivariate logistic regression model suggested that the following factors were significantly associated with CT infection: unmarried (AOR = 1.33, 95% CI = 1.02–1.74), cluster 2 (AOR = 2.54, 95% CI = 1.83–3.53), and cluster 4 (AOR = 3.31, 95% CI = 2.37–4.61). Using cluster 2 and cluster 4 as an indicator of CT can find 66.05% (179/271) CT positive cases, but the number of tests accounted for only 42.98% (1,090/2,536) of total male participants. Also, there was no difference between symptomatic and asymptomatic MSM.

**Table 2:** Univariate and multivariate logistic regression analyses of factors associated with NG in men.

**Variables**	**N (%)**	**Univariate OR (95% CI)**	***P***	**Multivariate AOR (95% CI)**	***P***
**Age group**	2,564 (100.00)				
>24	344 (13.42)	1			
≤24	2,220 (86.58)	2.75 (1.88–4.00)	<0.01		
**Marital status**	2,563 (100.00)				
Married	891 (34.76)	1		1	
Unmarried	1672 (65.24)	2.57 (1.84–3.60)	<0.01	1.89 (1.32–2.70)	<0.01
**Education**	2540 (100.00)				
Bachelor's degree or above	549 (21.57)	1			
Junior college	518 (20.39)	1.06 (0.62–1.82)	0.83		
High school/secondary technical school	782 (30.79)	1.43 (0.90–2.29)	0.13		
Junior high school or below	692 (27.24)	1.02 (0.61–1.69)	0.94		
**Having casual sexual partners in the** **last 3 months**	2,540 (100.00)				
No	1,186 (46.69)	1		1	
Yes	1,354 (53.31)	2.34 (1.62–3.37)	<0.01	2.03 (1.37–2.99)	<0.01
**STI test history**	2,521 (100.00)				
Yes	235 (9.32)	1			
No	2,286 (90.68)	1.16 (0.63–2.12)	0.64		
**Cluster model**	2,536 (100.00)				
Asymptomatic	1,271 (50.12)	1		1	
Cluster 1	85 (3.35)	2.53 (0.56–11.48)	0.23	2.97 (0.65–13.70)	0.16
Cluster 2	597 (23.54)	6.34 (3.26–12.33)	<0.01	6.70 (3.36–13.35)	<0.01
Cluster 3	90 (3.55)	3.62 (1.00–13.06)	0.05	3.27 (0.89–12.02)	0.07
Cluster 4	493 (19.44)	26.03 (14.14–47.91)	<0.01	24.53 (12.96–46.44)	<0.01

**Table 3:** Univariate and multivariate logistic regression analyses of factors associated with CT in men.

**Variables**	**N (%)**	**Univariate OR (95% CI)**	***P***	**Multivariate AOR (95% CI)**	***P***
**Age group**	2,564 (100.00)				
>24	344 (13.42)	1			
≤24	2,220 (86.58)	1.55 (1.12–2.15)	<0.01		
**Marital status**	2,563 (100.00)				
Married	891 (34.76)	1		1	
Unmarried	1,672 (65.24)	1.45 (1.12–1.87)	<0.01	1.33 (1.02–1.74)	0.04
**Education**	2,540 (100.00)				
Bachelor's degree or above	549 (21.57)	1			
Junior college	518 (20.39)	1.38 (0.92–2.08)	0.12		
High school/secondary technical school	782 (30.79)	1.42 (0.97–2.07)	0.07		
Junior high school or below	692 (27.24)	1.52 (1.04–2.23)	0.03		
**Having casual sexual partners in the last 3 months**	2,540 (100.00)				
No	1,186 (46.69)	1			
Yes	1,354 (53.31)	1.22 (0.95–1.58)	0.12		
**STI test history**	2,521 (100.00)				
Yes	235 (9.32)	1			
No	2,286 (90.68)	1.23 (0.77–1.97)	0.38		
**Cluster model**	2,536 (100.00)				
Asymptomatic	1,271 (50.12)	1		1	
Cluster 1	85 (3.35)	1.37 (0.61–3.08)	0.44	1.29 (0.54–3.06)	0.57
Cluster 2	597 (23.54)	2.57 (1.86–3.56)	<0.01	2.54 (1.83–3.53)	<0.01
Cluster 3	90 (3.55)	1.29 (0.58–2.88)	0.53	1.26 (0.56–2.82)	0.58
Cluster 4	493 (19.44)	3.56 (2.58–4.91)	<0.01	3.31 (2.37–4.61)	<0.01

### Women

#### Cluster analysis in women

Among female attendees, five clusters with distinct symptomatic patterns and a group of asymptomatic patients were identified, and 0.79% (45/5,671) and 8.31% (471/5,671) of the participants were diagnosed with gonorrhea and chlamydia, respectively. The frequency of gonorrhea and chlamydia in each cluster is shown in Table 4. The asymptomatic group accounted for 36.47% (2,068/5,671) of the total cohort, and 0.34 and 7.64% of the participants were diagnosed with gonorrhea and chlamydia, respectively. Cluster 1 consisted of 5.40% (306/5,671) of the entire cohort, including (1) urgency/urodynia/urethral swelling, redness or pus overflow, and (2) lower abdominal pain, and 0.33 and 7.84% of the participants were diagnosed with gonorrhea and chlamydia, respectively. Cluster 2 consisted of 1.64% (93/5,671) of the entire cohort, including (1) cervical congestion/mucus or purulent secretions, and 10.75% of the participants were diagnosed with chlamydia. Cluster 3 consisted of 41.10% (2,331/5,671) of the entire cohort, including (1) vaginal secretion increase or odors and (2) vaginal itching, and 1.33 and 8.67% of the participants were diagnosed with gonorrhea and chlamydia, respectively. Cluster 4 consisted of 1.68% (95/5,671) of the entire cohort, including (1) genital/perianal watery blisters or pustules, (2) genital/perianal ulcers, and (3) genital/perianal warts, and 1.05 and 8.42% of the participants were diagnosed with gonorrhea and chlamydia, respectively. Cluster 5 consisted of 13.72% (778/5,671) of the entire cohort, including (1) abnormal leucorrhoea and (2) inguinal lymph node enlargement, and 0.64 and 8.87% of the participants were diagnosed with gonorrhea and chlamydia, respectively.

**Table 4:** Symptom clusters of the respondents in the women cohort (*N* = 5,671).

**Symptoms present**	**Asymptomatic** ***n =* 2,068**	**Cluster 1** ***n =* 306**	**Cluster 2** ***n =* 93**	**Cluster 3** ***n =* 2331**	**Cluster 4** ***n =* 95**	**Cluster 5** ***n =* 778**
Vaginal secretion increase or odors				2,258		
Urgency/ urodynia/urethral swelling and redness or pus overflow		136				
Abnormal leucorrhoea						776
Cervical congestion/ mucus or purulent secretions			93			
Lower abdominal pain		188				
Genital / Perianal watery blisters or pustules					27	
Genital / Perianal ulcers					22	
Genital / Perianal warts					69	
Vaginal itching				80		
Inguinal lymph node enlargement						3
NG positive	7	1	0	31	1	5
CT positive	158	24	10	202	8	69

#### Factors associated with NG/CT infection in women

In univariate analyses, four variables were associated with NG/CT infection in women at *P* < 0.05 (shown in Tables 5, 6). Results from the multivariate logistic regression model suggested that the following factors were significantly associated with NG infection: aged 24 years or below (AOR = 6.27, 95% CI = 3.46–11.34) and cluster 3 (AOR = 3.68, 95% CI = 1.61–8.39). Using cluster 3 as an indicator of NG can find 63.27% (31/49) NG positive cases, but the number of tests accounted for only 41.10% (2,331/5,671) of total female participants. Results from the multivariate logistic regression model suggested that the following factors were significantly associated with CT infection: aged 24 years or below (AOR = 1.53, 95% CI = 1.16–2.01), unmarried (AOR = 2.08, 95% CI = 1.62–2.68), high school/secondary technical school (AOR = 1.52, 95% CI = 1.08–2.14), and junior high school or below (AOR = 1.82, 95% CI = 1.31–2.54).

**Table 5:** Univariate and multivariate logistic regression analyses of factors associated with NG in women.

**Variables**	**N (%)**	**Univariate OR (95% CI)**	***P***	**Multivariate AOR (95% CI)**	***P***
**Age group**	5,703 (100.00)				
>24	838 (14.69)	1		1	
≤24	4,865 (85.31)	7.09 (3.95–12.73)	<0.01	6.27 (3.46–11.34)	<0.01
**Marital status**	5,692 (100.00)				
Married	4,524 (79.48)	1			
Unmarried	1,168 (20.52)	3.93 (2.20–7.03)	<0.01		
**Education**	5,668 (100.00)				
Bachelor's degree or above	905 (15.97)	1			
Junior college	1,202 (21.21)	1.89 (0.59–6.04)	0.28		
High school/secondary technical school	1,623 (28.63)	2.53 (0.85–7.49)	0.09		
Junior high school or below	1,938 (34.19)	1.64 (0.54–4.99)	0.38		
**Having casual sexual partners in** **the last 3 months**	5,651 (100.00)				
No	4,036 (71.42)	1			
Yes	1,615 (28.58)	1.47 (0.81–2.68)	0.21		
**STI test history**	5,548 (100.00)				
Yes	435 (7.84)	1			
No	5,113 (92.16)	1.88 (0.45–7.78)	0.38		
**Cluster model**	5,671 (100.00)				
Asymptomatic	2,068 (36.47)	1		1	
Cluster 1	306 (5.40)	0.97 (0.12–7.87)	0.97	1.04 (1.13–8.52)	0.97
Cluster 2	93 (1.64)	<0.01 (< 0.01–>999.99)	0.99	<0.01 (< 0.01–>999.99)	0.98
Cluster 3	2,331 (41.10)	3.97 (1.74–9.03)	<0.01	3.68 (1.61–8.39)	<0.01
Cluster 4	95 (1.68)	3.13 (0.38–25.72)	0.29	2.40 (0.29–19.97)	0.42
Cluster 5	778 (13.72)	1.90 (0.60–6.02)	0.27	1.90 (0.60–6.03)	0.28

**Table 6:** Univariate and multivariate logistic regression analyses of factors associated with CT in women.

**Variables**	***n* (%)**	**Univariate OR (95% CI)**	**P**	**Multivariate AOR (95% CI)**	**P**
**Age group**	5,703 (100.00)				
>24	838 (14.69)	1		1	
≤24	4,865 (85.31)	2.44 (1.97–3.04)	<0.01	1.53 (1.16–2.01)	<0.01
**Marital status**	5,692 (100.00)				
Married	4,524 (79.48)	1		1	
Unmarried	1,168 (20.52)	2.51 (2.06–3.06)	<0.01	2.08 (1.62–2.68)	<0.01
**Education**	5,668 (100.00)				
Bachelor's degree or above	905 (15.97)	1		1	
Junior college	1,202 (21.21)	1.40 (0.99–2.00)	0.06	1.32 (0.92–1.89)	0.14
High school/secondary technical school	1,623 (28.63)	1.57 (1.13–2.19)	<0.01	1.52 (1.08–2.14)	<0.01
Junior high school or below	1,938 (34.19)	1.76 (1.28–2.42)	<0.01	1.82 (1.31–2.54)	<0.01
**Having casual sexual partners in the last 3 months**	5,651 (100.00)				
No	4,036 (71.42)	1			
Yes	1,615 (28.58)	1.11 (0.90–1.36)	0.34		
**STI test history**	5,548 (100.00)				
Yes	435 (7.84)	1		1	
No	5,113 (92.16)	1.69 (1.10–2.60)	0.02	1.55 (1.00–2.39)	0.05
**Cluster model**	5,673 (100.00)				
Asymptomatic	2,068 (36.47)	1			
Cluster 1	306 (5.40)	1.03 (0.66–1.61)	0.90		
Cluster 2	93 (1.64)	1.46 (0.74–2.86)	0.27		
Cluster 3	2,331 (41.10)	1.15 (0.92–1.43)	0.22		
Cluster 4	95 (1.68)	1.11 (0.53–2.34)	0.78		
Cluster 5	778 (13.72)	1.18 (0.88–1.58)	0.28		

## Discussion

This is the first study in China to explore the use of different symptom clusters as an indicator to identify NG and CT infection in STI-related clinics, providing new insight into symptomatic management. Symptomatic management and screening for both NG and CT based on symptom clusters were recommended for men presenting symptom clusters 2 and 4, including urethral syndrome (e.g., serous or purulent discharge and frequent urination/urgency/urodynia/itching), genital/perianal warts, balanitis, and inguinal lymph node enlargement, which was consistent with findings from previous studies and the WHO guideline (16–19). Using these two clusters as an indicator of testing can identify 88.59% of NG-positive cases and 66.05% of CT-positive cases, respectively. For women, we found that those with increased vaginal discharge, odor, and itching (cluster 3) had a higher risk of NG infection and were recommended to be tested for gonorrhea infection. Using this cluster as an indicator of testing can identify 63.27% of NG-positive cases. Similar results about vaginal discharge syndrome (e.g., vaginal discharge increases or odor and itching) were reported in a previous study (20). However, we suggested that symptomatic management was not suitable for both NG and CT infection in women in the Chinese context, which was inconsistent with the WHO guideline (16), but it still provided thoughts about NG/CT case finding in women, such as promoting opportunistic screening based on symptoms, age, and risk behaviors. We also identified several non-symptom risk factors, and the inclusion of risk assessment in symptomatic management was beneficial in reducing over-treatment rates and increasing correct treatment rates and was cost-effective (21).

According to this study, if laboratory testing is available, men with urethral discharge syndromes, genital/perianal warts, balanitis, and inguinal lymph node enlargement are recommended to take the NAAT test for NG/CT infection. If laboratory tests are not available, symptomatic management could be used for men with the above symptoms for NG and CT to ensure same-day treatment. Risk factors of NG (e.g., unmarried and having casual sexual partners in the last 3 months) and CT (e.g., unmarried) in men found in this study could also be considered in symptomatic management or screening with higher accuracy. Clusters including abnormal discharge and frequent urination/urgency/urodynia/itching were found to be an indicator of NG/CT infection, and these clusters belong to urethral discharge syndromes, which was consistent with the WHO guideline and a previous study (16, 19). Similarly, the guidelines revealed that the odds of NG or CT infection among men with urethral discharge is 10 times the odds among men with no urethral discharge (16). Other studies also reported that urethral discharge syndromes can be a diagnostic symptom for the NG and CT in men, which was suggested as a cost-effective strategy (5, 22). Similarly, the performance of urethral discharge syndromes for NG and CT infections has been adequate in other studies as well (23, 24). The guidelines also recommended that if the urethral discharge is present but tests are negative, treatment for non-gonococcal and non-chlamydial urethritis (such as *Mycoplasma genitalium* or *Trichomonas vaginalis*) should be considered (16). Also, similar findings about other important but relatively unusual symptoms were shown in previous studies. A study showed that among those with a confirmed NG or CT visit, balanitis was observed in 24% of cases and genital warts in 12% of cases (25). Lymphogranuloma venereum (LGV) is caused by CT, and 22.5% of infections had symptoms of inguinal lymph node enlargement (26). Therefore, it would be useful to link these symptoms with NG and CT when offering symptomatic management. NAAT is relatively expensive and not available everywhere, especially in some resource-limited places or rural areas, so primary care settings with many patients with urethral discharge syndromes may face challenges. Antigen detection and point-of-care-test (POCT) are cheap and convenient. For NG and CT, the sensitivity and specificity of the POCT assay were more than 90%, and it can facilitate testing and reduce missed patients, which is recommended in resource-limited places (27).

We suggest that symptomatic management based on vaginal discharge syndrome is not highly effective in detecting NG in women. Meanwhile, NG is highly resistant and should not be treated for symptoms alone (28), and NAAT or other accurate tests are needed to confirm the diagnosis. Therefore, vaginal discharge syndrome along with risk factors for NG (e.g., aged 24 years or below) could be considered an indicator of NG screening. The results of this study showed that diagnosis based on vaginal discharge syndrome (vaginal secretion increase or odors and vaginal itching) could detect 63.27% of NG cases in women, but with low sensitivity. Studies have shown that cervicovaginal cytokine concentrations did not differ between women with asymptomatic STIs and those with symptomatic STIs (29). Symptomatic treatment based on vaginal discharge is more accurate in the population with a high prevalence of NG and CT (female sexual workers, etc.), with a positive predictive value of more than 27% but less accurate in the general population (30, 31). Most vaginal discharge abnormalities are due to vaginal infections caused by *Trichomonas vaginalis* and bacterial vaginosis rather than cervical infections caused by NG and CT (32). In Morocco, a study showed that the prevalence of NG was 0.37% and CT was 3.8% among those with vaginal discharge syndrome (20). Therefore, we do not recommend treatment of NG in the general population of women based on symptoms alone.

Symptomatic management is not suitable for CT infection in women in the Chinese context as none of the symptoms were associated with CT infection, which was inconsistent with the WHO guideline (16). The results of this study showed that women infected with CT have no specific symptoms. One possible reason for the above situation was that more than 85% of CT infections in women are asymptomatic, and asymptomatic infections can last for months (33). Risk factors for CT (e.g., aged 24 years or below, unmarried, high school/secondary technical school, and junior high school or below) in women found in this study could be considered in CT screening with higher accuracy. For health authorities, it may be better to focus on the improvement of the laboratory capability in China rather than promote symptomatic management based on STI symptoms. During the COVID-19 pandemic in China, any patients with a fever seeking healthcare in secondary and tertiary hospitals needed to be screened by polymerase chain reaction (PCR) testing (34), which improved the capability of laboratories in these hospitals, so how to integrate NAAT testing for NG and CT infection into these hospitals should also be considered.

The prevalence of NG and CT was 5.23 and 13.21%, respectively. A surprising finding in this study was that 9.79% (19/194) of NG and 31.81% (236/742) of CT infections were asymptomatic, which would have been missed if using symptomatic management. Although the proportion of asymptomatic patients in this study was lower than in other studies due to the recruitment of clinical patients, all patients should be screened if accurate testing for NG/CT is available. Asymptomatic infection is a burden in the control of sexually transmitted diseases because it can serve as a bridge population in the transmission of NG/CT. Similar to symptomatic infection, asymptomatic infection also leads to adverse outcomes and affects people's reproductive health, which calls for an urgent need for interventions for this population. The result of this study indicated that having casual sexual partners in the last 3 months, aged 24 years or below, and having lower education were sociodemographic and behavioral factors of NG/CT infections, which could be considered in the expansion of routine screening in the general population. Promoting health literacy and sexually transmitted infection education is important because most people lacked an understanding of NG and CT infections, and having a correct understanding of the outcome of CT infection was associated with high screening willingness (35).

There were some limitations to the study. First, the follow-up data of positive cases was not collected, which should be considered in future studies. Second, the sample size of MSM in this study was not large enough for analyses, and more research is needed to further explore the differences between symptomatic and asymptomatic MSM. Third, information related to sexual behaviors was self-reported, which may lead to social desirability bias.

## Conclusion

This study found that NG and CT symptomatic management was recommended for men but not for women. Sociodemographic and behavioral factors of NG/CT infections, including having had casual sexual partners in the last 3 months, being aged 24 years or below, and having lower education, could be integrated into symptomatic management and considered in the expansion of routine screening.

## Data availability statement

The datasets presented in this article are not readily available because of patient privacy. Requests to access the datasets should be directed to the corresponding author.

## Ethics statement

The studies involving human participants were reviewed and approved by Ethical Review Committee of the Shenzhen Center for Chronic Disease Control (Approval No. 20180206). The patients/participants provided their written informed consent to participate in this study.

## Author contributions

NN and YC conceived and designed the study. NN, RW, CZ, LW, JY, HW, and JL supervised the data collection. YC, XC, RW, CZ, LW, JY, HW, JL, and NN performed the research. NN, RW, and YC analyzed and interpreted the results and were the major contributors in writing the manuscript. XC, CZ, LW, JY, HW, and JL revised the manuscript critically. All authors read and approved the final manuscript.

## Funding

This study was supported by Sanming project of Medicine in Shenzhen [Grant No. SZSM201611077].

## Conflict of interest

The authors declare that the research was conducted in the absence of any commercial or financial relationships that could be construed as a potential conflict of interest.

## Publisher's note

All claims expressed in this article are solely those of the authors and do not necessarily represent those of their affiliated organizations, or those of the publisher, the editors and the reviewers. Any product that may be evaluated in this article, or claim that may be made by its manufacturer, is not guaranteed or endorsed by the publisher.
